# Small vessel disease and cognitive reserve oppositely modulate global network redundancy and cognitive function: A study in middle‐to‐old aged community participants

**DOI:** 10.1002/hbm.26634

**Published:** 2024-03-29

**Authors:** Lei Cui, Hui Hong, Shuyue Wang, Qingze Zeng, Yeerfan Jiaerken, Xinfeng Yu, Ruiting Zhang, Yao Zhang, Linyun Xie, Miao Lin, Lingyun Liu, Xiao Luo, Kaicheng Li, Xiaocao Liu, Jixuan Li, Peiyu Huang, Minming Zhang

**Affiliations:** ^1^ Department of Radiology The Second Affiliated Hospital of Zhejiang University, School of Medicine Hangzhou China

**Keywords:** brain network, cerebral small vessel disease, cognitive reserve, dynamic functional connectome, redundancy, resilience

## Abstract

Cerebral small vessel disease (SVD) can disrupt the global brain network and lead to cognitive impairment. Conversely, cognitive reserve (CR) can improve one's cognitive ability to handle damaging effects like SVD, partly by optimizing the brain network's organization. Understanding how SVD and CR collectively influence brain networks could be instrumental in preventing cognitive impairment. Recently, brain redundancy has emerged as a critical network protective metric, providing a nuanced perspective of changes in network organization. However, it remains unclear how SVD and CR affect global redundancy and subsequently cognitive function. Here, we included 121 community‐dwelling participants who underwent neuropsychological assessments and a multimodal MRI examination. We visually examined common SVD imaging markers and assessed lifespan CR using the Cognitive Reserve Index Questionnaire. We quantified the global redundancy index (RI) based on the dynamic functional connectome. We then conducted multiple linear regressions to explore the specific cognitive domains related to RI and the associations of RI with SVD and CR. We also conducted mediation analyses to explore whether RI mediated the relationships between SVD, CR, and cognition. We found negative correlations of RI with the presence of microbleeds (MBs) and the SVD total score, and a positive correlation of RI with leisure activity‐related CR (CRI‐leisure). RI was positively correlated with memory and fully mediated the relationships between the MBs, CRI‐leisure, and memory. Our study highlights the potential benefits of promoting leisure activities and keeping brain redundancy for memory preservation in older adults, especially those with SVD.

## Graphical abstract

Understanding how SVD and CR collectively impact the global network is pivotal in preventing cognitive impairment. SVD negatively correlates with global network redundancy, while CR positively correlates. Redundancy fully mediates the SVD, CR, and memory relationship, emphasizing redundancy's role in preserving memory in older adults, especially those with SVD.

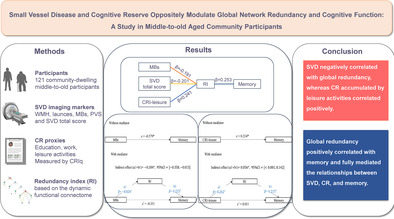

## 1 INTRODUCTION

Cerebral small vessel disease (SVD) is a chronic, progressive disorder mainly affecting the brain's small perforating arterioles, capillaries, and venules (Wardlaw et al., 2019, 2022). It is common in individuals over 60 (de Leeuw et al., 2001) and is increasingly recognized as a significant vascular contributor to approximately 45% of global dementia cases (METACOHORTS Consortium, 2016; Wardlaw et al., 2019). Many studies suggest that SVD contributes to cognitive decline partially by disrupting the brain's network, including widespread network disconnections, impaired small‐world properties, and reduced efficiency (Dey et al., 2016; Frey et al., 2021; Ter Telgte et al., 2018; Xin et al., 2022).

Conversely, modifiable factors like education can positively impact cognition partially by optimizing network organization and enhancing efficiency (Chan et al., 2021; Kim et al., 2021). Other lifelong experiences may yield similar benefits (Chan et al., 2018; Cheng, 2016; Soldan et al., 2021). In this context, cognitive reserve (CR) becomes essential. CR suggests that exposure to diverse and rich lifetime experiences can be a comprehensive protective factor, encouraging brain enrichment and resilience to neurological damage, thereby benefiting cognition (Stern et al., 2020; Stern et al., 2023). Higher levels of CR may counteract the harmful effects of conditions like SVD (Murray et al., 2011; Pinter et al., 2015; Vemuri et al., 2015).

Understanding how SVD and CR collectively influence the brain network is pivotal in preventing cognitive impairment. Existing studies have effectively examined the focal frontoparietal control network backbones (Chen et al., 2022; Ye et al., 2022). However, they may not comprehensively capture the collective network effect of SVD and CR.

Recently, brain network redundancy has emerged as a potential metric. This metric considers backup elements within network (Avena‐Koenigsberger et al., 2017), such as weaker connections that help preserve network functionality under damage (Avena‐Koenigsberger et al., 2017; Gallos et al., 2012; Rajkumar et al., 2022). Greater redundancy suggests improved resilience and functional maintenance (Glassman, 1987; Navlakha et al., 2014; Tononi et al., 1999) and has been linked to better preservation of specific cognitive functions (Langella, Mucha, et al., 2021; Langella, Sadiq, et al., 2021; Sadiq et al., 2021). Given that SVD can disrupt global functional networks from an early stage (Ter Telgte et al., 2018), it is reasonable that SVD could also disrupt global redundancy, leading to a loss of the network's capacity to preserve overall functionality. Conversely, lifespan CR‐promoting causes may improve such redundancy, as suggested by a structural network study (Wook Yoo et al., 2015), which may mitigate the network impact of SVD.

Currently, less is known about the specific cognitive function related to global redundancy, and how SVD and CR affect this redundancy and afterward cognition. We aim to explore (1) the associations between global redundancy and specific cognitive performances, (2) the associations of this redundancy with SVD and CR, and (3) whether global redundancy is a mediator between SVD, CR, and cognition in the community‐dwelling participants.

## 2 MATERIALS AND METHODS

### 2.1 Participants

We retrospectively examined our community‐based cohort, formed through random screening of participants in Hangzhou, China. These participants were recruited via local advertisements from 2019 onwards. All participants provided written informed consent prior to the study. The Medical Ethics Committee of the Second Affiliated Hospital of Zhejiang University School of Medicine approved the research protocols. All procedures adhered to the principles of the Declaration of Helsinki.

Inclusion criteria included: (1) age ≥ 45 years; (2) complete multimodal magnetic resonance imaging (MRI) data, including T1‐weighted (T1), T2‐weighted (T2), T2 fluid‐attenuated inversion recovery (T2‐FLAIR) images, susceptibility‐weighted (SWI) images, and resting‐state functional MRI (rs‐fMRI) images; and (3) complete neuropsychological assessments and the Cognitive Reserve Index questionnaire (CRIq) (Nucci et al., 2012). Based on these criteria, we included 131 participants.

Exclusion criteria included: (1) severe neurological or psychiatric disorders, dementia or family history of hereditary neurodegenerative diseases, brain trauma, or systemic diseases with significant brain impact (8 participants excluded); (2) severe head motion (mean framewise displacement (FD) > 0.5 mm or max FD >5 mm or over 10% outlier data points) during MRI scanning (Power et al., 2014) (1 participant excluded); (3) poorly registered functional atlas (1 participant excluded).

### 2.2 Demographic information and neuropsychological assessment

Demographic and clinical information was collected from all subjects, including age, sex, smoking status, diabetes mellitus, hypertension, hyperlipidemia, and hyperhomocysteinemia. Smoking status was determined according to the subjects' self‐report. Diabetes mellitus was defined as the presence of fasting serum glucose >7.0 mmol/L or postprandial 2 h plasma glucose >11.1 mmol/L or having a previous history of diabetes. Hypertension was defined as systolic blood pressure ≥140 mmHg or diastolic pressure ≥90 mmHg measured twice in quiet conditions or having a self‐reported history of hypertension. Hyperlipidemia was defined as self‐reported hyperlipidemia, or treatment with anti‐dyslipidemia medication, fasting total cholesterol >5.2 mmol/L, or low‐density lipoprotein >3.36 mmol/L. Hyperhomocysteinemia was defined as the presence of fasting plasma total cysteine concentration >15 mmol/L or having a self‐reported history of hyperhomocysteinemia.

Neuropsychological assessments were conducted across various domains. Global cognition was assessed using the Montreal Cognitive Assessment and the mini‐mental state examination. Memory was evaluated with the auditory verbal learning test. Language ability was measured using the Boston Naming Test. Processing speed and executive function were assessed using the Trail Making Test part A and part B, respectively. Attention was evaluated using the digit symbol substitution test and the digit span test (DS). Each cognitive test score was transformed into a standardized Z‐score. Further details are available in Supplementary Materials Table 1.

### 2.3 CR assessment

We assessed CR for all participants using the CRIq (Nucci et al., 2012), conducted by trained researchers. The CRIq is a tool that collects demographic data, education years, lifetime occupational and leisure activities. Each of these elements independently and distinctively contributes to CR (Foubert‐Samier et al., 2012). The questionnaire provides individual scores for three domains (CRI‐Education, CRI‐Work, CRI‐Leisure) and a cumulative CRI‐total score.

### 2.4 MR acquisition

All MR images were acquired using a United Imaging MR790 3.0 T scanner. T1 images were acquired with a 3D fast spoiled gradient echo sequence; the parameters were TR = 6.9 ms, TE = 2.9 ms, flip angle = 9°, TI = 1000 ms, field of view (FOV) = 256 × 240 mm, voxel size = 1 × 1 × 1 mm, and 208 sagittal slices. T2 images were acquired with a modulated flip angle technique in refocused imaging with an extended echo train (MATRIX) sequence; the parameters were TR = 3000 ms, TE = 405.46 ms, echo train length = 180, FOV = 256 × 240 mm, voxel size = 0.8 × 0.8 × 0.8 mm, and 208 sagittal slices. T2‐FLAIR images were acquired with inversion recovery MATRIX sequence; the parameters were TR = 6500 ms, TE = 432.48 ms, TI = 1938 ms, echo train length = 220, echo spacing = 4.24 ms, FOV = 256 × 220 mm, voxel size = 1 × 1 × 1 mm, and 170 sagittal slices. The SWI sequence used a three‐dimension multi‐echo gradient‐echo sequence with 8 equally spaced echoes: echo time = 4.5 ms [first echo], inter‐echo spacing = 4.5 ms, repetition time = 34 ms, FOV = 24 cm × 24 cm, matrix size = 416 × 384, flip angle = 20°, slice thickness = 2.0 mm with no gap between slices, and in‐plane spatial resolution of 0.4688 mm/pixel × 0.4688 mm/pixel. The rs‐fMRI scanning was performed in darkness, and the participants were explicitly instructed to relax and not fall asleep during acquisition. The rs‐fMRI scan was conducted for a total of 8 min and 11 s, using an echo‐planar imaging sequence. The acquisition parameters were TR = 2000 ms, TE = 30 ms, voxel size = 3.5 mm, flip angle = 70°, number of slices = 38.

### 2.5 Visual assessment of SVD markers

Two neuroradiologists blinded to clinical information independently assessed SVD visual markers, including white matter hyperintensities (WMH), dilation of perivascular spaces (PVS), lacunes, and microbleeds (MBs) according to the STRIVE guidelines (Wardlaw et al., 2013).

WMH was evaluated separately for periventricular and deep regions using the Fazekas scale (Fazekas et al., 1987), with grades ranging from 0 (absent) to 3 (irregular extensions into subcortical white matter for periventricular; confluent for deep). PVS dilation, identified as a cerebrospinal fluid‐like signal smaller than 3 mm without a hyperintense rim, was assessed on 5 mm axial reconstructions of T2 images for compatibility with prior studies. Basal ganglia and deep white matter were each rated on a scale from 0 (none) to 4 (≥ 40 PVS). Lacunes were identified on T2‐FLAIR images as round or ovoid fluid‐filled cavities, 3–15 mm in diameter, consistent with a previous acute subcortical infarct or hemorrhage. MBs were recorded as small, signal void areas with associated blooming on SWI, excluding signal voids from sulcal vessels, calcifications, choroid plexus, and low‐signal averaging from adjacent bone.

Moreover, we compiled the SVD total score (Staals et al., 2014), assigning one point for any of the following: one or more lacunars, one or more MBs, moderate to severe BG‐PVS (grades 2–4), periventricular WMH Fazekas 3 or deep WMH Fazekas 2–3. A neuroimaging researcher (P.H.) reviewed all results and resolved any disputes to finalize the ratings.

### 2.6 Rs‐fMRI preprocessing and network construction

The rs‐fMRI data were processed using the functional MRI PREProcessing pipeline, fMRIprep v20.20.0 (https://fmriprep.org/), with default processing steps (Esteban et al., 2019). Specifically, each T1 image underwent correction for intensity non‐uniformity and skull‐stripping. Volume‐based spatial normalization to the ICBM 152 Nonlinear Asymmetrical template version 2009c was executed through nonlinear registration, employing brain‐extracted versions of the T1 images and the template. Tissue‐type segmentation was performed on the brain‐extracted T1 images. Functional data were subjected to corrections for slice‐timing, head motion, and field distortion. Following these steps, co‐registration to the corresponding T1 images was carried out using boundary‐based registration with six degrees of freedom, and potential confounds of interest were extracted.

Subsequently, fMRIdenoise (https://github.com/compneuro-ncu/fmridenoise) was utilized to denoise all processed functional data with the 24HMP8PhysSpikeReg pipeline (Power et al., 2012; Satterthwaite et al., 2013). The confound regression incorporated 24 head motion parameters (three translations, three rotations, their temporal derivatives, and quadratic terms), eight physiological noise parameters (mean signals from white matter and cerebrospinal fluid, their temporal derivatives, and quadratic terms), and spike regressors based on mean FD and DVARS thresholds. Following these steps, all functional data were resampled to 3 mm isotropic, smoothed with a 5 mm full width at half maximum Gaussian kernel, and masked by gray matter.

We then employed the sliding window methods, based on dynamicBC (https://www.nitrc.org/projects/dynamicbc/), to analyze the denoised signal (Liao et al., 2014). Specifically, a window length of 30 volumes (60 s) with a step size of one volume (2 s) was utilized, creating a 58 s overlap between any two consecutive windows (Leonardi & Van De Ville, 2015). Within each window, we computed functional connectivity matrices as the Pearson's correlations (Fisher's *r*‐to‐*z* transformed) between the BOLD signal time courses of every pair of regions in the 268‐node whole‐brain Shen functional parcellation scheme (Shen et al., 2013). To conduct a more comprehensive analysis of the dFC, we further applied binarization using proportional thresholding across multiple densities (van den Heuvel et al., 2017).

### 2.7 Redundancy calculation

**FIGURE 1:**
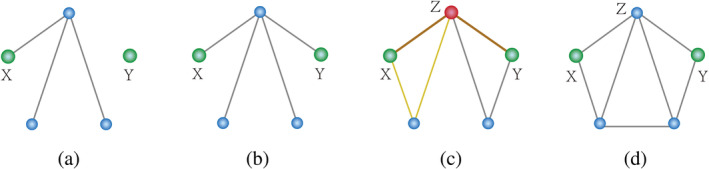
Illustration of connected and two‐connected networks. (A) Disconnected network: Lack of connection in the network halts information flow from node X to Y. (B) Connected but not two‐connected network: Although information can flow from X to Y, any damage along the pathway breaks this transmission, indicating limited robustness. (C) Another connected but not two‐connected network: Despite improved robustness over network B and potential for info flow from X to Y, damage to node Z still halts transmission. (D) Two‐connected network: This robust network maintains uninterrupted information flow from X to Y, despite any damage to a node or edge along the pathway.

In line with prior studies (Ghanbari, Soussia, et al., 2022), we assessed dynamic functional redundancy by assessing the network's propensity to display robust topological structure during scanning. To this end, we adopted two network metrics—connectedness and two‐connectedness. Connectedness indicates the degree to which all nodes in a brain network are interconnected, with a path existing between each pair of nodes and no isolated nodes. Two‐connectedness builds upon this concept by ensuring that at least two distinct paths exist between each pair of nodes, not sharing any nodes other than themselves, thus more robust (please see details in Figure 1).

Such metrics are heavily dependent on network density. As the density increases, the brain network transitions from disconnected to connected and then to two‐connected, with critical points marking these transitions. Following previous studies, we employed density thresholds of 5%–50% to mitigate saturation effects in 5% increments, creating 10 binary networks per sliding window. We defined redundancy states based on whether the minimally connected network was also two‐connected, sidestepping the consideration of two‐connected transition points for each sliding window. We assigned a metric index of “1” if the minimally connected network was also two‐connected and “0” otherwise. We then quantified the redundancy index (RI) by calculating the occurrence probability of the “1” index across all sliding windows.

A higher RI value suggests a greater likelihood for the network to preserve its functionality, which may benefit cognition.

### 2.8 Statistical analysis

Statistical analyses were performed using IBM SPSS Statistics 26, R version 4.2.2 and Python 3.1.2. The Kolmogorov–Smirnov test was used to examine the normality of the data distribution.

#### 2.8.1 The associations of cognition with RI, SVD, and CR

Linear regression models were used to explore the associations of specific cognitive domains with RI, SVD, and CR. Each cognitive performance was set as a distinct dependent variable. Age, sex, and years of education were included as covariates. However, when certifying the associations of cognition with CRI‐education and CRI‐total score, only age and sex were used as covariates, given that the influence of education was already accounted for in these two proxies (Nucci et al., 2012). To account for multiple testing, *p*‐values were further adjusted with the R package “p.adjust” function according to the method of Benjamini/Hochberg (B/H) to control the false discovery rate (FDR) in each individual cognitive domain. An association was considered to be statistically significant, if its corresponding B/H‐adjusted *p*‐value was below .05, corresponding to an FDR of 5%.

#### 2.8.2 The associations of RI with SVD and CR

Linear regression models were used to explore whether CR and SVD could predict RI. In Model 1, each SVD marker and CR domain was analyzed individually with RI as the outcome variable. Model 2 integrated all variables, applying a stepwise (forward and backward) selection to find the factor that could predict RI independently. In both models, age, sex, and mean FD were included as covariates. Model 3 followed the same method as Model 2, but expanded the covariates to incorporate vascular risk factors, including smoking status, hypertension, diabetes, hyperlipidemia, and hyperhomocysteinemia, considering their potential confounding effects on the SVD or RI. To avoid overfitting and highly inflated estimation of R‐squared in stepwise regression, we also conducted 10‐fold cross‐validation with Python.

#### 2.8.3 The mediation effects of RI between SVD, CR, and cognition

Based on our assumptions, we conducted mediation analyses to explore whether RI mediated the relationships between SVD, CR, and cognition. This analysis was carried out using PROCESS version 4.0 (https://processmacro.org/index.html) in SPSS. We evaluated whether the total effect (*c*) of SVD burden and CR on cognition is attributable to a significant direct effect (*c*′) or is instead mediated by indirect effects (*ab*) through the mediator variable. A bootstrapping approach with 5000 iterations was utilized to estimate confidence intervals (CI). Effects were considered significant if the bootstrapped 95% CI did not include zero.

In all the above regression models, we checked for multicollinearity using the variance inflation factor, with a threshold of ≤10 indicating acceptable collinearity. The standardized beta coefficients indicated the predictive power of each variable.

### 2.9 Validation analysis

Considering the potential effect of the sliding window lengths on the results, we calculated RI at different window lengths (40 and 50 volumes). We then repeated all regression and mediation analyses to ensure the robustness of our findings (please see Supplementary Materials (Data S1)).

## 3 RESULTS

### 3.1 Demographics

One hundred twenty‐one participants were included. Table 1 shows the demographics, CR domains, SVD markers, cognitive performance, and RI of included subjects.

**TABLE 1:** Characteristics of the subjects.

	*N* = 121
Demographics	
Age, y, median (IQR)	57.5 (54.1–63.2)
Female, *n* (%)	69 (57.0)
Education, y, median (IQR)	9.0 (6.0–10.5)
Smoking, *n* (%)	33 (27.3)
Diabetes, *n* (%)	11 (9.1)
Hypertension, *n* (%)	49 (40.5)
Hyperlipemia, *n* (%)	21 (17.4)
Hyperhomocysteinemia, *n* (%)	39 (32.2)
CR domains	
CRI‐education, mean (SD)	90.5 (2.7)
CRI‐work, median (IQR)	97.0 (89.8–112.3)
CRI‐leisure, median (IQR)	79.0 (71.8–86.3)
CRI‐total score, median (IQR)	85.5 (76.8–94.3)
SVD imaging markers	
Presence of lacunes, *n* (%)	12 (9.9)
Presence of MBs, *n* (%)	12 (9.9)
dwPVS, median (IQR)	1 (1–2)
bgPVS, median (IQR)	1 (1–1)
F: peri WM, median (IQR)	1 (0–1)
F: deep WM, median (IQR)	1 (1–1)
SVD total score, median (IQR)	0 (0–1)
Cognitive performance	
MMSE, median (IQR)	28 (27–29)
MOCA, mean (SD)	22.73 (3.9)
DS, median (IQR)	12 (10–13)
DSST, median (IQR)	41 (32–48)
TMT‐A, median (IQR)	62.9 (50.1–80.2)
TMT‐B, median (IQR)	162.2 (124.1–210.1)
AVLT_ immediate recall, mean (SD)	15.4 (4.4)
AVLT_ delayed recall, mean (SD)	9.7 (4.4)
AVLT_ recognition, median (IQR)	22 (20–23)
BNT, median (IQR)	23 (20–26)
Functional imaging metric	
Mean FD, median (IQR)	0.11 (0.08–0.14)
RI, percentage, mean (SD)	48.0 (7.3)

*Note*: Statistical results are reported as mean (standard deviation, SD) if the original data follows a normal distribution or median (interquartile range, IQR). For TMT‐A and TMT‐B, lower values indicate higher performance. For all other cognitive measures, higher values indicate higher performance.

Abbreviations: AVLT, auditory verbal learning test; bgPVS, basal ganglia perivascular space; BNT, Boston naming test; CRI, cognitive reserve index; dwPVS, deep white matter perivascular space; DS, digit span test; DSST, digit symbol substitution test; F: peri, Fazekas score for periventricular white matter; F: deep WM, Fazekas score for deep white matter; MBs, microbleeds; MMSE, Mini Mental State Exam; Moca, Montreal Cognitive Assessment; Mean FD, mean framewise displacement; TMT‐A and TMT‐B, Trail Making Test part A and part B; RI, redundancy index.

### 3.2 The associations of cognition with RI, SVD, and CR

Table 2 shows that after controlling for age, sex, and years of education, RI was only positively associated with the memory domain (*β* = .253, *p* = .003).

**TABLE 2:** The associations of cognition with RI, SVD, and CR.

	Global cognition		Memory		Language		Processing speed		Executive function		Attention	
	Std.β	*p*	Std.β	*p*	Std.β	*p*	Std.β	*p*	Std.β	*p*	Std.β	*p*
CR domains												
CRI‐education	.526	<.001**	.350	<.001**	.579	<.001**	.287	.001**	.346	<.001**	.447	<.001**
CRI‐work	.098	.239	.184	.044*	.123	.107	.118	.177	.060	.484	.179	.024*
CRI‐leisure	.020	.801	.214	.016**	.082	.271	.089	.304	.128	.126	.059	.455
CRI‐total score	.476	<.001**	.446	<.001**	.544	<.001**	.363	<.001**	.390	<.001**	.482	<.001**
SVD imaging markers												
F: peri WM	−.004	.955	.084	.330	−.010	.894	.071	.393	−.042	.604	.009	.901
F: deep WM	−.055	.469	.161	.057	.049	.488	.044	.589	−.035	.664	−.039	.601
Presence of lacunes	−.151	.050*	.035	.691	−.064	.371	−.028	.738	−.029	.719	.051	.501
Presence of MBs	−.098	.186	−.170	.040*	−.089	.196	.069	.384	.048	.532	.016	.826
dwPVS	.007	.925	.029	.740	.037	.602	−.075	.350	−.093	.242	−.083	.261
bgPVS	−.099	.206	−.068	.444	.080	.269	.003	.974	−.014	.866	.040	.600
SVD total score	−.174	.027*	−.034	.704	−.015	.835	.071	.396	−.024	.777	.052	.506
Network metric												
RI	.031	.685	.253	.003**	−.014	.843	−.032	.692	−.008	.924	−.007	.930

*Note*: Values depicted were standardized coefficients (Std.β) from linear regression models with each cognitive performance as a dependent variable and CR, SVD, and RI as the independent variable separately. Age, sex, and years of education were used as covariates. However, when analyzing cognition associations with CRI‐education and CRI‐total score, only age and sex were considered as covariates.

Abbreviations: bgPVS, basal ganglia perivascular space; CRI, cognitive reserve index; dwPVS, deep white matter perivascular space; F: peri, Fazekas score for periventricular white matter; F: deep WM, Fazekas score for deep white matter; MBs, microbleeds; RI, redundancy index.

* *p*‐value <.05.

** Benjamini/Hochberg—adjusted *p*‐value <.05.

For SVD markers, the presence of lacunes (*β* = −.151, *p* = .050) and the SVD total score (*β* = −.174, *p* = .027) were negatively associated with global cognition. The presence of MBs was negatively associated with memory (*β* = −.170, *p* = .040). No significant associations were found among other SVD imaging markers and cognition domains.

For CR domains, CRI‐education and CRI‐total score were positively associated with all six cognitive domains. CRI‐work was positively associated with memory (*β* = .184, *p* = .044) and attention (*β* = .179, *p* = .024), while CRI‐leisure was positively associated only with memory (*β* = .214, *p* = .016).

After B/H correction, most associations retained their significance. However, the associations of cognition with CRI work and SVD imaging markers (both SVD total score and the presence of MBs) did not survived. B/H‐adjusted *p*‐values were reported in the Supplementary Table 2.

### 3.3 The associations of RI with SVD and CR

As shown in Table 3, in Model 1 after controlling for age, sex, and mean FD, CRI‐leisure was positively associated with RI (*β* = .241, *p* = .005). Negative associations were observed with the SVD total score (*β* = −.201, *p* = .037) and the presence of MBs (*β* = −.191, *p* = .029). In Model 2, which employed a stepwise selection method, CRI‐leisure (*β* = .245, *p* = .004) and the presence of MBs (*β* = −.197, *p* = .020) could individually predict RI. Model 3, which incorporated vascular risk factors as covariates, remains selected CRI‐leisure (*β* = .224, *p* = .008) and the presence of MBs (*β* = −.187, *p* = .031) unchanged. This model exhibited an adjusted R‐squared value of 0.2113, and a mean squared error (MSE) value of 41.9956.

**TABLE 3:** The associations of RI with SVD and CR.

	Model 1			Model 2			Model 3		
	Std.β	95%CI	*p*	Std.β	95%CI	*p*	Std.β	95%CI	*p*
CR domains									
CRI‐education	.018	−0.094, 0.115	.843						
CRI‐work	.111	−0.032, 0.137	.217						
CRI‐leisure	.241	0.043, 0.242	.005*	.245	0.048, 0.243	.004*	.224	0.035, 0.230	.008*
CRI‐total score	.147	−0.014, 0.164	.096						
SVD imaging markers									
F: peri WM	−.139	−3.782, 0.489	.129						
F: deep WM	−.074	−3.128, 1.303	.416						
Presence of lacunes	−.142	−7.834, 0.931	.121						
Presence of MBs	−.191	−8.786, −0.494	.029*	−.197	−8.803, −0.772	.020*	−.187	−8.655, −0.422	.031*
dwPVS	−.104	−2.547, 0.687	.254						
bgPVS	−.130	−4.285, 0.709	.159						
SVD total score	−.201	−3.248, −0.100	.037*						

*Note*: Values depicted were standardized coefficients (Std.β) from linear regression models with RI as the dependent variable and CR, SVD as the independent variable. Model 1 conducted univariate regression for each predictor individually, adjusting for age, sex, and mean FD. Model 2 integrated all variables, applying stepwise selection to find those could predict RI independently, also controlling for age, sex, and mean FD. Model 3 followed the stepwise method employed in Model 2, expanded the covariates to incorporate vascular risk factors, including smoking status, hypertension, diabetes, hyperlipidemia, and hyperhomocysteinemia.

Abbreviations: bgPVS, basal ganglia perivascular space; CRI, cognitive reserve index; dwPVS, deep white matter perivascular space; F: peri, Fazekas score for periventricular white matter; F: deep WM, Fazekas score for deep white matter; s, microbleeds; RI, redundancy index.

* *p*‐value <.05.

Following the implementation of 10‐fold cross‐validation, the stepwise regression model consistently selected CRI‐leisure and presence of MBs as the predictive variables. The model exhibited an increased adjusted R‐squared of 0.2387, and a similar MSE value of 42.8912.

### 3.4 RI fully mediates the MBs’ and CRI‐leisure’ effect on memory

Given the distinct correlation of RI with memory, coupled with the distinct correlations of both MBs and CRI‐leisure with RI and memory, we specifically examined if RI could mediate the associations of MBs, CRI‐leisure, and memory.

**FIGURE 2:**
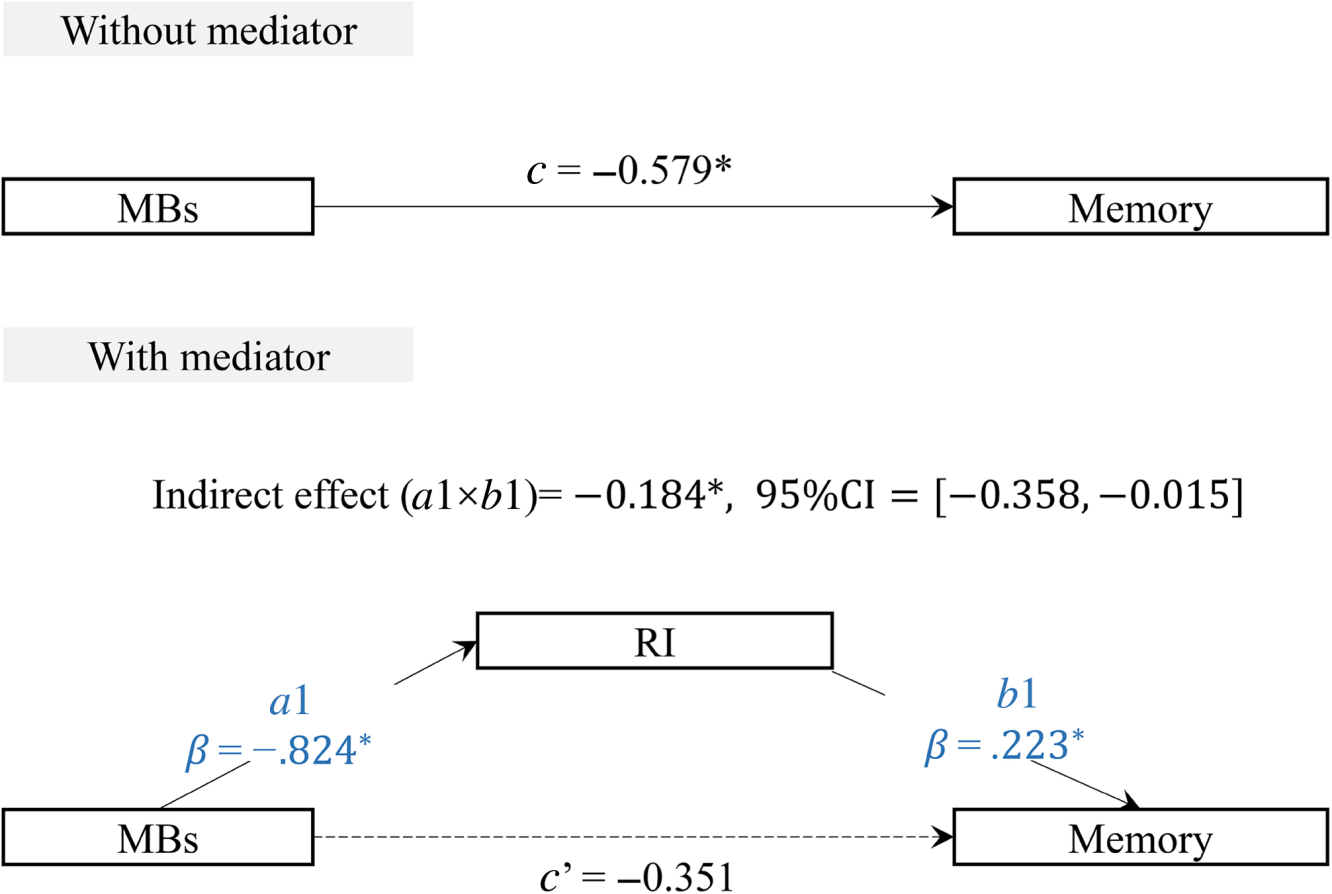
Mediation analysis of RI between the presence of MBs and memory. The effect of MBs on memory is shown by the direct effect [*c*] without and the indirect effect [*c*′] with the mediator. Standardized β‐coefficients of each path [*a* and *b*] are shown for the mediator [*p < .05]. Significant paths are indicated by solid arrows, and nonsignificant paths are shown by dashed arrows. Indirect effects are statistically significant at the 95% CI when the CI does not include 0. As the direct effect [*c*′] is not significant, RI fully mediates the relationship between presence of MBs and memory [this analysis controlled for age, sex and education]. CI, confidence interval; MBs, microbleeds; RI, redundancy index.

**FIGURE 3:**
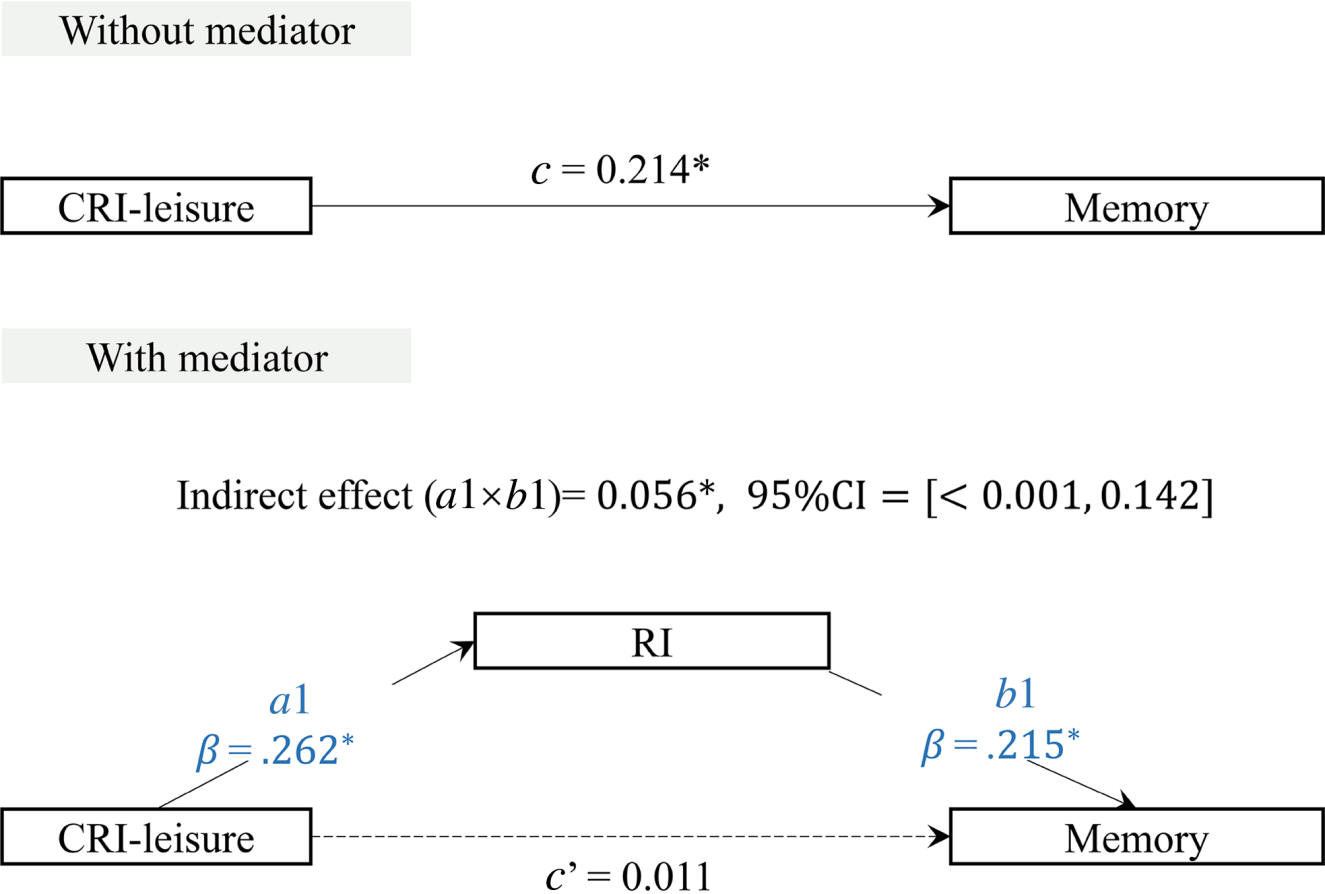
Mediation analysis of RI between CRI‐leisure and memory. The effect of CRI‐leisure on memory is shown by the direct effect [*c*] without and the indirect effect [*c*′] with the mediator. Standardized β‐coefficients of each path [*a* and *b*] are shown for the mediator [*p < .05]. Significant paths are indicated by solid arrows, and nonsignificant paths are shown by dashed arrows. Indirect effects are statistically significant at the 95% CI when the CI does not include 0. As the direct effect [*c*′] is not significant, RI fully mediates the relationship between CRI‐leisure and memory [this analysis controlled for age, sex and education]. CI, confidence interval; CRI, cognitive reserve index; RI, redundancy index.

As Figures 2 and 3 illustrate, the mediation analysis revealed a significant indirect effect of the presence of MBs (*a*1 × *b*1 = −0.184, 95% CI = [−0.358, −0.015]) and CRI‐leisure (*a*1 × *b*1 = 0.056, 95% CI = [<0.001, 0.142]) on memory via RI. The direct effect of MBs (*c*′ = −0.351, *p* > .05) and CRI‐leisure (*c*′ = 0.011, *p* > .05) on memory was not significant after adjusting for the mediator, supporting full mediation models. These results suggest that RI fully mediates the relationship between the presence of MBs, CRI leisure and memory.

### 3.5 Validation analysis

The validation analyses using different window sizes (40 and 50 volumes) confirmed the robustness of our primary findings (please see Supplementary Materials (Data S1)).

## 4 DISCUSSION

In this study, we explored the specific cognitive domain related to global redundancy and how SVD, CR affect this redundancy and subsequently cognition in a community cohort. Our findings suggested that global redundancy is positively associated with memory. SVD negatively correlated with this redundancy, whereas CR accumulated through leisure activities correlated positively. Global redundancy fully mediates the deteriorating effect of MBs and the protective effect of leisure activities related to CR on memory. These findings underscore the potential of promoting leisure activities and keeping robust brain redundancy as strategies for preserving memory in older adults, particularly for those with SVD.

We validated that network redundancy is positively associated with cognition, specifically the memory domain, corroborating other human and animal brain studies (Chen et al., 2021;Langella, Mucha, et al., 2021; Langella, Sadiq, et al., 2021;Sadiq et al., 2021). Given the understanding that memory is a distributed function encompassing the entire cortical system and relies on efficient communication and coordination among various cortical regions (Hasson et al., 2015), it is reasonable that a well‐structured global redundancy, which signifies a more robust and interconnected neural network, could benefit memory performance.

Our findings revealed a negative correlation between global redundancy with both the SVD total score and the presence of MBs. Considering SVD's tendency to directly inflict damage on the white matter and trigger widespread disconnection (Ter Telgte et al., 2018), the network's capacity to preserve functionality might be compromised. Besides, a previous study has noted a reduction in global redundancy during the final two decades of life (Sadiq et al., 2021). Coupled with our findings that SVD independently affects redundancy, it raises the possibility that the growing prevalence of SVD could contribute to the observed reduction in redundancy during aging.

Our results illustrate a positive correlation between lifetime leisure activities and global redundancy. A previous research (Wook Yoo et al., 2015) using structural network analysis has identified that education can enhance the redundancy of focal networks. Our study did not replicate this finding from a global perspective. It is plausible that the networks impacted by education are more confined (Chan et al., 2021), while leisure activities may significantly contribute to global topology from a broad scope (Soldan et al., 2021).

The importance of RI for memory is further emphasized by the result that RI fully mediates the effect of MBs and CRI‐leisure on memory. Other studies have found that redundancy mediates the effect of brain atrophy on memory (Langella, Mucha, et al., 2021). Therefore, tracking this metric within community groups may be helpful, considering the impact of pathology and leisure activities on redundancy. Such tracking may provide insights into mitigating memory decline.

While SVD affects multiple cognitive domains, especially processing speed and executive function (Hamilton et al., 2021), we only found damage in the global cognition and memory domain. However, these results did not withstand correction for multiple comparisons. This difference could be attributed to our community subjects' relatively mild SVD burden and the potential lack of sensitivity in some cognitive tests. In addition, ours and other redundancy‐related studies (Langella, Mucha, et al., 2021; Langella, Sadiq, et al., 2021; Sadiq et al., 2021) found no significant correlation between redundancy with processing speed and executive function. These two cognitive domains may rely more on direct shortest connections. Once such connections are damaged, the matching cognition may be affected, and the other redundant paths cannot compensate for the slow information transmission. Many studies have found that WMH and lacunes damage the global network (Frey et al., 2021; Ter Telgte et al., 2018), but we did not find these affect global redundancy. The mechanism behind this requires further investigation.

To our best knowledge, this is the first study to investigate the collective effect of SVD and CR from a global perspective. Given that SVD is a whole‐brain disorder that can provoke widespread disconnections even in early pathology (Ter Telgte et al., 2018; Wardlaw et al., 2019), our global perspective is particularly well‐suited to study related network changes. Since our study focuses on community‐dwelling populations, it offers findings with more applicability and may offer new insights for further studies. The use of the CRIq allowed us to examine various cognitive stimuli activities. This study also leverages the advantages of dynamic redundancy, including its robust definition, dynamic connectivity, and heightened sensitivity (Ghanbari et al., 2023; Ghanbari, Soussia, et al., 2022; Ghanbari, Zhou, et al., 2022).

This study has several limits. The cross‐sectional design limits our ability to detect dynamic changes and establish causality over time, indicating a need for future longitudinal studies. Our sample size and a relatively younger population with a lower SVD burden may constrain the statistical power. Future studies could explore these associations in larger samples and among populations with a higher SVD burden. Although we tried to mitigate the potential influences of other neurodegenerative conditions on redundancy, we should admit that we cannot erase the possibility of external interference. Future studies might consider incorporating indicators such as amyloid‐β and α‐synuclein to rule out potential influences from diseases like Alzheimer's disease and Parkinson's disease. Additionally, research has indicated that resting‐state connectivity demonstrates reduced stability when participants are in a closed‐eye state rather than an eyes‐open, fixated state (Patriat et al., 2013). The lack of a visual stimulation device within our MRI setup is a major limitation of our study. We recommend that future studies incorporate such considerations to enhance the stability of resting‐state connectivity findings.

## 5 CONCLUSION

A well‐structured network of global redundancy is associated with more preserved memory performance among community‐dwelling participants. SVD negatively correlates with this redundancy, whereas CR accumulated through lifelong leisure activities shows a positive correlation. Brain redundancy fully mediates the associations between SVD, CR, and memory.

## FUNDING INFORMATION

This work was supported by the National Natural Science Foundation of China (Grant No: 81971577; 82371907; 82101987; 81901706; 82101984; 82271936; 82202090; 82302138) and the Natural Science Foundation of Zhejiang Province (Grant No: LQ20H180015), China Postdoctoral Science Foundation (Grant No: 2019M662083), and Zhejiang traditional Chinese medicine Youth Talent Fund project (Grant No: 2022ZQ057).

## CONFLICT OF INTEREST STATEMENT

The authors have declared that no competing interests exist.

## Supporting information


**Data S1** Supporting information

## Data Availability

The data that support the findings of this study are available on request from the corresponding author. The data are not publicly available due to privacy or ethical restrictions.
