# Supplementary material for: Small vessel disease and cognitive reserve oppositely modulate global network redundancy and cognitive function: A study in middle‐to‐old aged community participants
Source: Hum Brain Mapp. 2024 Mar 29;45(5):e26634. doi: 10.1002/hbm.26634 (PMC10980841; doi:10.1002/hbm.26634)
Supplement: Supplementary file 1 — Data S1 Supporting information [file HBM-45-e26634-s001.docx]

**Supplementary Materials**

**Supplementary Table 1. Numbers of subjects completed cognitive assessments**

| Cognitive assessments | Function / Total participants |
| --- | --- |
| MMSE | Global cognition (N=121) |
| MoCA | Global cognition (N=120) |
| AVLT | Memory (N=116) |
| BNT | Language (N=120) |
| DSST | Attention (N=117) |
| DS | Attention (N=120) |
| TMT-A | Information processing speed (N=120) |
| TMT-B | Executive function (N=117) |

Abbreviations: MMSE, Mini Mental State Exam; Moca, Montreal Cognitive Assessment; DS, Digit Span Test; AVLT, Auditory Verbal Learning Test; TMT-A and TMT-B, Trail Making Test part A and part B; DSST, Digit Symbol Substitution Test; BNT, Boston Naming Test.

**Supplementary Table 2. The associations of cognition with RI, SVD, and CR with adjusted p-values according to Benjamini/Hochberg**

|  | Global cognition | |  | Memory | |  | Language | |  | Processing speed | | | |  | | Executive function | | | |  | | Attention | | | |  | |  |
| --- | --- | --- | --- | --- | --- | --- | --- | --- | --- | --- | --- | --- | --- | --- | --- | --- | --- | --- | --- | --- | --- | --- | --- | --- | --- | --- | --- | --- |
|  | Std.β | BH-adj p |  | Std.β | BH-adj p |  | Std.β | BH-adj p |  | Std.β | | BH-adj p | |  | | Std.β | | BH-adj p | |  | | Std.β | | BH-adj p | |  | |  |
| **CR domains** | | | | | | | | | | | | | | | | | | | | | | | | | | | |  |
| CRI-education | 0.526 | <0.001* |  | 0.350 | 0.001* |  | 0.579 | <0.001* |  | 0.287 | | 0.006* | |  | | 0.346 | | 0.001* | |  | | 0.447 | | <0.001* | |  | |  |
| CRI-work | 0.098 | 0.410 |  | 0.184 | 0.088 |  | 0.123 | 0.428 |  | 0.118 | | 0.594 | |  | | 0.060 | | 0.924 | |  | | 0.179 | | 0.096 | |  | |  |
| CRI-leisure | 0.020 | 0.955 |  | 0.214 | 0.048* |  | 0.082 | 0.542 |  | 0.089 | | 0.594 | |  | | 0.128 | | 0.504 | |  | | 0.059 | | 0.801 | |  | |  |
| CRI-total score | 0.476 | <0.001* |  | 0.446 | <0.001* |  | 0.544 | <0.001* |  | 0.363 | | 0.001* | |  | | 0.390 | | <0.001* | |  | | 0.482 | | <0.001* | |  | |  |
| **SVD imaging markers** | | | | | | | | | | | | | | | | | | | | | | | | | | | |  |
| F: peri WM | -0.004 | 0.955 |  | 0.084 | 0.495 |  | -0.010 | 0.894 |  | | 0.071 | | 0.594 | |  | | -0.042 | | 0.924 | |  | | 0.009 | | 0.930 | |  | |
| F: deep WM | -0.055 | 0.704 |  | 0.161 | 0.098 |  | 0.049 | 0.732 |  | | 0.044 | | 0.785 | |  | | -0.035 | | 0.924 | |  | | -0.039 | | 0.801 | |  | |
| Presence of lacunes | -0.151 | 0.150 |  | 0.035 | 0.740 |  | -0.064 | 0.636 |  | | -0.028 | | 0.805 | |  | | -0.029 | | 0.924 | |  | | 0.051 | | 0.801 | |  | |
| Presence of MBs | -0.098 | 0.410 |  | -0.170 | 0.088 |  | -0.089 | 0.542 |  | | 0.069 | | 0.594 | |  | | 0.048 | | 0.924 | |  | | 0.016 | | 0.930 | |  | |
| dwPVS | 0.007 | 0.955 |  | 0.029 | 0.740 |  | 0.037 | 0.803 |  | | -0.075 | | 0.594 | |  | | -0.093 | | 0.726 | |  | | -0.083 | | 0.783 | |  | |
| bgPVS | -0.099 | 0.410 |  | -0.068 | 0.592 |  | 0.080 | 0.542 |  | | 0.003 | | 0.974 | |  | | -0.014 | | 0.924 | |  | | 0.040 | | 0.801 | |  | |
| SVD total score | -0.174 | 0.108 |  | -0.034 | 0.740 |  | -0.015 | 0.894 |  | | 0.071 | | 0.594 | |  | | -0.024 | | 0.924 | |  | | 0.052 | | 0.801 | |  | |
| **Network metric** | | | | | | | | | | | | | | | | | | | | | | | | | | | |  |
| RI | 0.031 | 0.913 |  | 0.253 | 0.012* |  | -0.014 | 0.894 |  | -0.032 | | 0.805 | |  | | -0.008 | | 0.924 | |  | | -0.007 | | 0.930 | |  | |  |

Note: Values depicted were standardized coefficients (Std.β) from linear regression models with each cognitive performance as a dependent variable while CR, SVD, and RI as the independent variable separately. Age, sex, and years of education were used as covariates. However, when analyzing cognition associations with CRI-education and CRI-total score, only age and sex were considered as covariates. * BH-adjusted p value < 0.05.

Abbreviations: CRI, cognitive reserve index; F: peri, Fazekas score for periventricular white matter; F: deep WM, Fazekas score for deep white matter; MBs, microbleeds; dwPVS, deep white matter perivascular space; bgPVS, basal ganglia perivascular space; RI, redundancy index; BH-adj p, adjusted p-values according to Benjamini/Hochberg.

**Supplementary Table 3. Validation Analysis: Relationships of cognitive performance with RI_40 and RI_50.**

|  | Global cognition | |  | Memory | |  | Language | |  | Processing speed | |  | Executive function | |  | Attention | |  |
| --- | --- | --- | --- | --- | --- | --- | --- | --- | --- | --- | --- | --- | --- | --- | --- | --- | --- | --- |
|  | Std.β | p |  | Std.β | p |  | Std.β | p |  | Std.β | p |  | Std.β | p |  | Std.β | p |  |
| RI_40 | 0.049 | 0.522 |  | 0.284 | 0.001* |  | 0.063 | 0.368 |  | -0.034 | 0.677 |  | -0.053 | 0.510 |  | -0.026 | 0.722 |  |
| RI_50 | 0.097 | 0.203 |  | 0.222 | 0.009* |  | 0.027 | 0.705 |  | -0.006 | 0.938 |  | -0.006 | 0.941 |  | -0.038 | 0.609 |  |

Note: Values depicted are standardized coefficients (Std.β) from linear regression models with each cognitive performance as a dependent variable and RI_40, RI_50 as the independent variable separately. Age, sex, and years of education were used as covariates. * p-value < 0.05.

Abbreviations: RI_40, redundancy index calculated using a window length of 40 volumes; RI_50, redundancy index calculated using a window length of 50 volumes

**Supplementary Table 4. Validation Analysis: Relationships of RI_40 with SVD and CR.**

| Variable | Model1 | | |  | Model2 | | |  | Model3 | | |
| --- | --- | --- | --- | --- | --- | --- | --- | --- | --- | --- | --- |
|  | Std.β | 95%CI | p |  | Std.β | 95%CI | p |  | Std.β | 95%CI | p |
| **CR domains** | | | | | | | |  |  |  |  |
| CRI-education | 0.067 | -0.070, 0.151 | 0.467 |  |  |  |  |  |  |  |  |
| CRI-work | 0.088 | -0.046, 0.134 | 0.333 |  |  |  |  |  |  |  |  |
| CRI-leisure | 0.208 | 0.022, 0.237 | 0.019* |  | 0.222 | 0.034, 0.243 | 0.010* |  | 0.196 | 0.016, 0.228 | 0.024* |
| CRI-total score | 0.146 | -0.016, 0.173 | 0.101 |  |  |  |  |  |  |  |  |
| **SVD imaging markers** | | | | | | | |  |  |  |  |
| F: peri WM | -0.105 | -3.579, 0.974 | 0.259 |  |  |  |  |  |  |  |  |
| F: deep WM | -0.109 | -3.764, 0.923 | 0.232 |  |  |  |  |  |  |  |  |
| Presence of lacunes | -0.170 | -9.000, 0.260 | 0.064 |  |  |  |  |  |  |  |  |
| Presence of MBs | -0.210 | -9.782, -0.961 | 0.017* |  |  |  |  |  | -0.211 | -9.896, -0.930 | 0.018* |
| dwPVS | -0.126 | -2.937, 0.515 | 0.167 |  |  |  |  |  |  |  |  |
| bgPVS | -0.145 | -4.749, 0.540 | 0.118 |  |  |  |  |  |  |  |  |
| SVD total score | -0.248 | -3.783, -0.574 | 0.008* |  | -0.262 | -3.868, -0.732 | 0.004* |  |  |  |  |

Note: Values depicted are standardized coefficients (Std.β) from linear regression models with RI_40 as the dependent variable and CR, SVD as the independent variable. Model 1 analyzed each independent variable individually. Model 2 integrated all variables by applying a stepwise selection method. Both model 1 and 2 included age, sex, and mean FD as covariates. Model 3 expanded the covariates to incorporate vascular risk factors, including smoking status, hypertension, diabetes, hyperlipidemia, and hyperhomocysteinemia. * p-value < 0.05.

Abbreviations: CRI, cognitive reserve index; F: peri, Fazekas score for periventricular white matter; F: deep WM, Fazekas score for deep white matter; MBs, microbleeds; dwPVS, deep white matter perivascular space; bgPVS, basal ganglia perivascular space; RI_40, redundancy index calculated using a window length of 40 volumes.

**Supplementary Table 5. Validation Analysis: Relationships of RI_50 with SVD and CR.**

| Variable | Model1 | | |  | Model2 | | |  | Model3 | | |
| --- | --- | --- | --- | --- | --- | --- | --- | --- | --- | --- | --- |
|  | Std.β | 95%CI | p |  | Std.β | 95%CI | p |  | Std.β | 95%CI | p |
| **CR domains** | | | | | | | |  |  |  |  |
| CRI-education | < 0.001 | -0.133, 0.133 | 0.997 |  |  |  |  |  |  |  |  |
| CRI-work | 0.036 | -0.087, 0.130 | 0.691 |  |  |  |  |  |  |  |  |
| CRI-leisure | 0.179 | 0.003, 0.263 | 0.045* |  | 0.193 | 0.016, 0.270 | 0.027* |  |  |  |  |
| CRI-total score | 0.083 | -0.061, 0.168 | 0.357 |  |  |  |  |  |  |  |  |
| **SVD imaging markers** | | | | | | | |  |  |  |  |
| F: peri WM | -0.063 | -3.673, 1.818 | 0.505 |  |  |  |  |  |  |  |  |
| F: deep WM | -0.077 | -4.018, 1.633 | 0.405 |  |  |  |  |  |  |  |  |
| Presence of lacunes | -0.121 | -9.292, 1.922 | 0.196 |  |  |  |  |  |  |  |  |
| Presence of MBs | -0.178 | -10.765, -0.085 | 0.047* |  |  |  |  |  | -0.182 | -10.951, -0.170 | 0.043* |
| dwPVS | -0.050 | -2.658, 1.520 | 0.590 |  |  |  |  |  |  |  |  |
| bgPVS | -0.192 | -6.473, -0.165 | 0.039* |  |  |  |  |  |  |  |  |
| SVD total score | -0.242 | -4.460, -0.594 | 0.011* |  | -0.254 | -4.556, -0.748 | 0.007* |  |  |  |  |

Note: Values depicted are standardized coefficients (Std.β) from linear regression models with RI_50 as the dependent variable and CR, SVD as the independent variable. Model 1 analyzed each independent variable individually. Model 2 integrated all variables by applying a stepwise selection method. Both model 1 and 2 included age, sex, and mean FD as covariates. Model 3 expanded the covariates to incorporate vascular risk factors, including smoking status, hypertension, diabetes, hyperlipidemia, and hyperhomocysteinemia. * p-value < 0.05.

Abbreviations: CRI, cognitive reserve index; F: peri, Fazekas score for periventricular white matter; F: deep WM, Fazekas score for deep white matter; MBs, microbleeds; dwPVS, deep white matter perivascular space; bgPVS, basal ganglia perivascular space; RI_50, redundancy index calculated using a window length of 50 volumes.

**
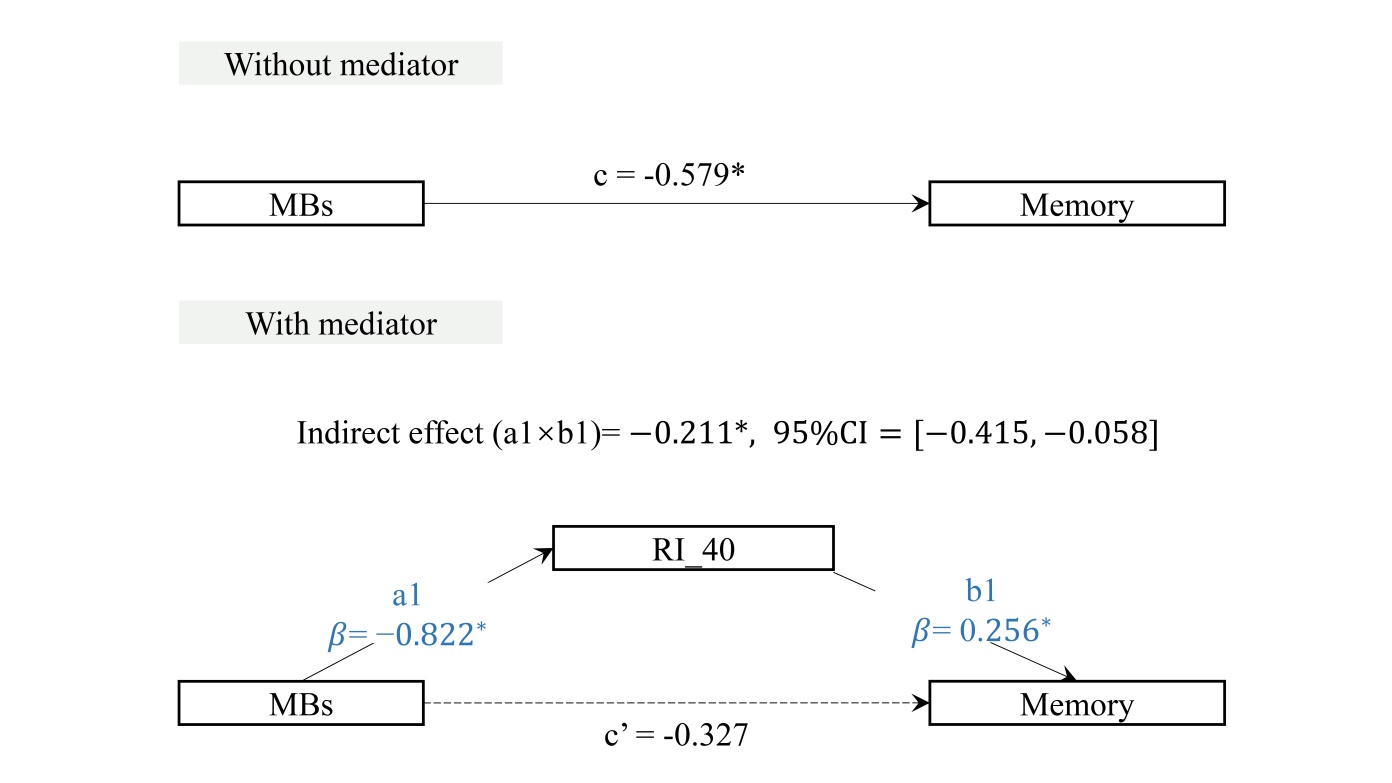
**

**Supplementary Figure 1. Validation Analysis: Mediation analysis of RI_40 between MBs and memory.** The effect of MBs on memory is shown by the direct effect [c] without and the indirect effect [c'] with the mediator. Standardized β‐coefficients of each path [a and b] are shown for the mediator [*p < .05]. Significant paths are indicated by solid arrows, and nonsignificant paths are shown by dashed arrows. Indirect effects are statistically significant at the 95% CI when the CI does not include 0. As the direct effect [c'] is not significant, RI_40 fully mediates the relationship between presence of MBs and memory [this analysis controlled for age, sex and education]. MBs, microbleeds; CI, confidence interval; RI_40, redundancy index calculated using a window length of 40 volumes. **
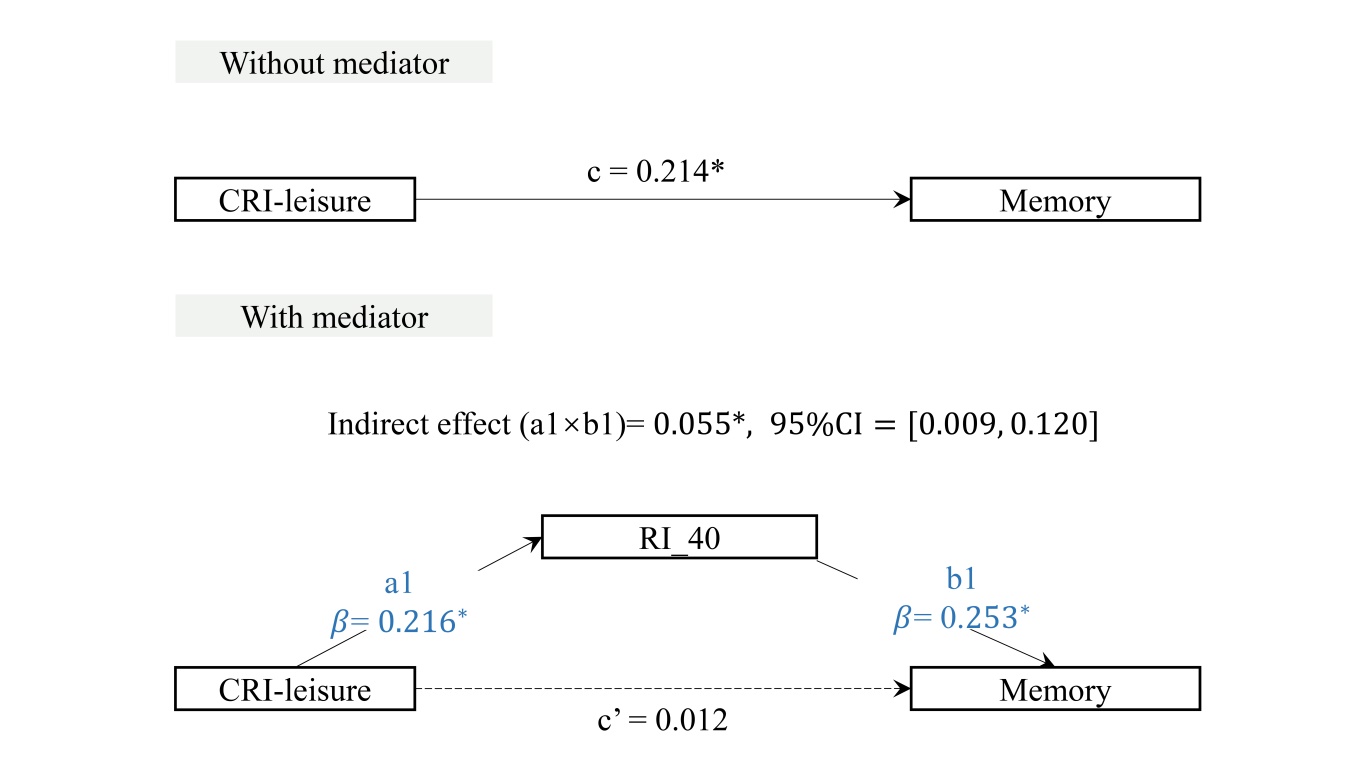
**

**Supplementary Figure 2. Validation Analysis: Mediation analysis of RI_40 between CRI-leisure and memory.** The effect of CRI-leisure on memory is shown by the direct effect [c] without and the indirect effect [c'] with the mediator. Standardized β‐coefficients of each path [a and b] are shown for the mediator [*p < .05]. Significant paths are indicated by solid arrows, and nonsignificant paths are shown by dashed arrows. Indirect effects are statistically significant at the 95% CI when the CI does not include 0. As the direct effect [c'] is not significant, RI_40 fully mediates the relationship between CRI-leisure and memory [this analysis controlled for age, sex and education]. CRI, cognitive reserve index; CI, confidence interval; RI_40, redundancy index calculated using a window length of 40 volumes.

**
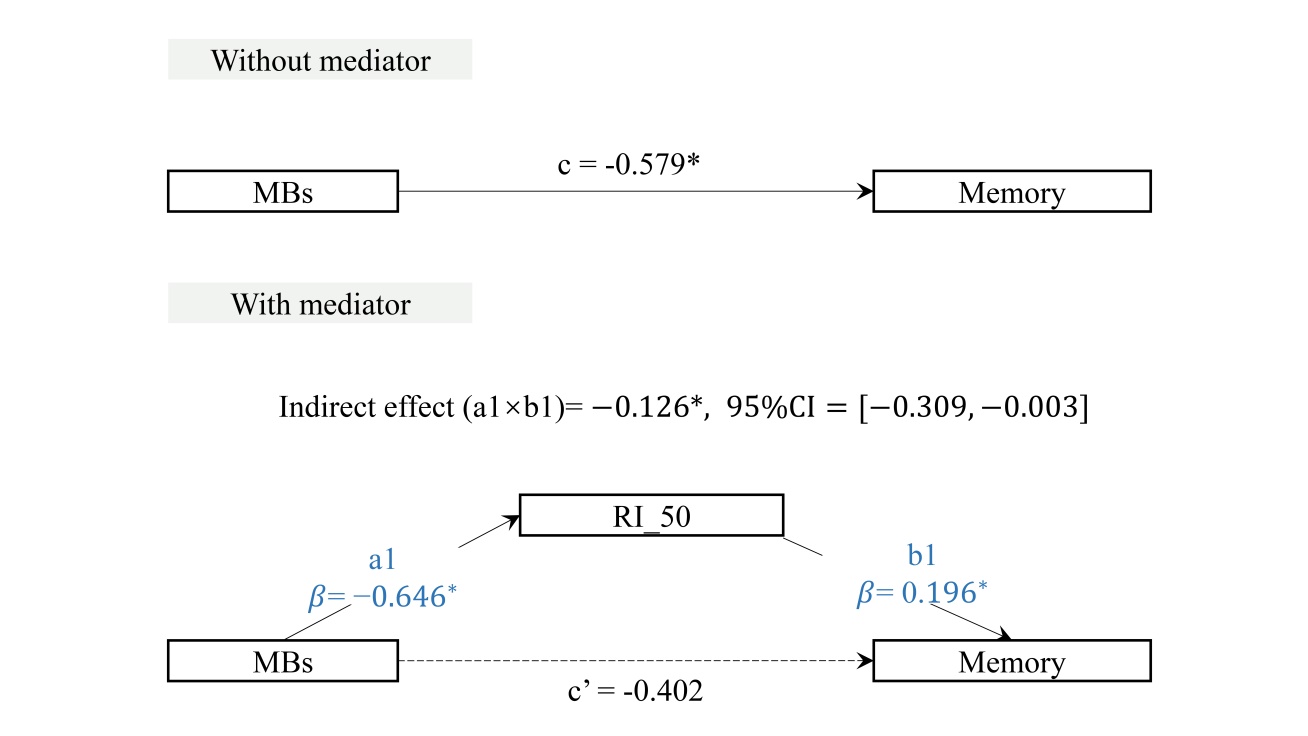
**

**Supplementary Figure 3. Validation Analysis: Mediation analysis of RI_50 between MBs and memory.** The effect of MBs on memory is shown by the direct effect [c] without and the indirect effect [c'] with mediator. Standardized β‐coefficients of each path [a and b] are shown for the mediator [*p < .05]. Significant paths are indicated by solid arrows, and nonsignificant paths are shown by dashed arrows. Indirect effects are statistically significant at the 95% CI when the CI does not include 0. As the direct effect [c'] is not significant, RI_50 fully mediates the relationship between presence of MBs and memory [this analysis controlled for age, sex and education]. MBs, microbleeds; CI, confidence interval; RI_50, redundancy index calculated using a window length of 50 volumes.

**
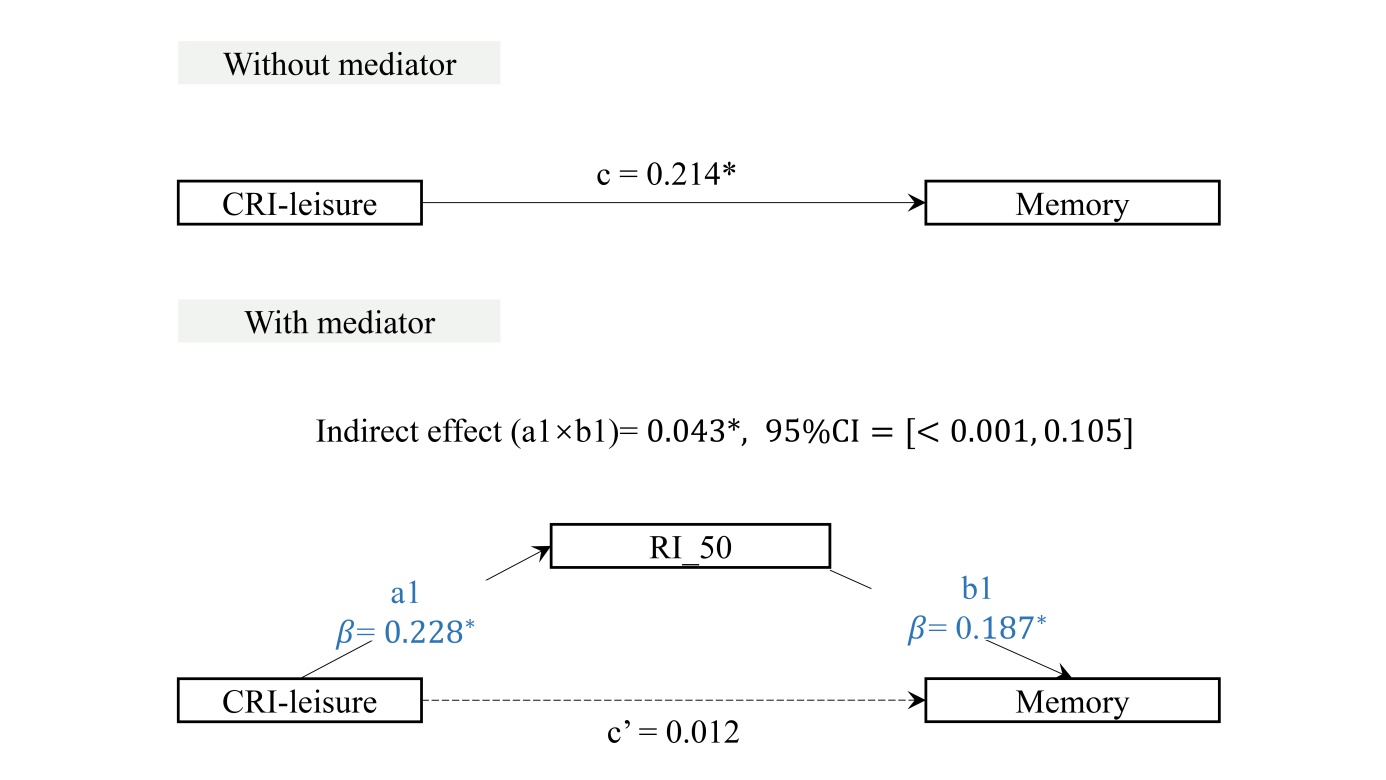
**

**Supplementary Figure 4. Validation Analysis: Mediation analysis of RI_50 between CRI-leisure and memory.** The effect of CRI-leisure on memory is shown by the direct effect [c] without and the indirect effect [c'] with the mediator. Standardized β‐coefficients of each path [a and b] are shown for the mediator [*p < .05]. Significant paths are indicated by solid arrows, and nonsignificant paths are shown by dashed arrows. Indirect effects are statistically significant at the 95% CI when the CI does not include 0. As the direct effect [c'] is not significant, RI_50 fully mediates the relationship between CRI-leisure and memory [this analysis controlled for age, sex and education]. CRI, cognitive reserve index; CI, confidence interval; RI_50, redundancy index calculated using a window length of 50 volumes.
